# Vascular calcification in different arterial beds in ex vivo ring culture and in vivo rat model

**DOI:** 10.1038/s41598-022-15739-w

**Published:** 2022-07-13

**Authors:** Karen Muyor, Jonas Laget, Irene Cortijo, Flore Duranton, Bernard Jover, Àngel Argilés, Nathalie Gayrard

**Affiliations:** 1 RD Néphrologie, 2 Rue des Mûriers, 34090 Montpellier, France; 2 BC2M, Université de Montpellier, Montpellier, France; 3 Néphrologie Dialyse Saint Guilhem (NDSG), Sète, France

**Keywords:** Chronic kidney disease, Calcification, Calcium and vitamin D

## Abstract

Vascular calcification is a risk factor for cardiovascular and kidney diseases. Medial calcification may differently affect the arterial tree depending on vessel location and smooth muscle injury. The aim was to map the anatomical distribution of vascular calcifications on different arteries and artery locations, in cultured artery rings (ex vivo) and in a rat model of elastocalcinosis (in vivo). Vascular calcification was assessed histologically (von Kossa staining of the media) and by calcium content measurement. Arteries of different sizes were harvested from untreated rats for ring culture and from the vitamin D_3_-nicotine (VDN) rat model for direct observation. When cultured in pro-calcifying conditions, thoracic aorta exhibited similar calcification from the arch to the diaphragm. Calcification increased in abdominal aorta along with the reduction in cross sectional area. Carotid and renal arteries exhibited similar ex vivo calcification. In VDN rats, calcification was greater in carotid artery than in aorta, and was accompanied by fibrosis and apoptosis. Ex vivo, calcification was increased by the induction of lesions on arteries. Along the vascular tree, calcification of the arterial wall increases with the narrowing of vessels in ex vivo ring culture and in vivo. The observed differences represent local susceptibility of the vessels to the calcifying processes.

## Introduction

Vascular calcification is observed in pathophysiological conditions in human associated to aging^1^, hypertension^2,3^, diabetes mellitus^4^ and chronic renal failure^5–8^. In turn, vascular calcification is a risk factor for cardiovascular^9^ and kidney diseases^5^. Medial calcification induces arterial wall stiffening and increases in pulse wave velocity, pulse pressure and systolic arterial pressure^10^, leading to both structural and functional impairments of the heart and peripheral organs^4,11^. It is now accepted that both, active and passive mechanisms participate in the pathophysiology of vascular calcification leading to calcium hydroxyapatite crystals deposition in the arterial wall^12,13^. Calcium and phosphate deposition involves vascular smooth muscle cells (VSMCs) trans-differentiation to osteoblast-like cells^14,15^ and necrosis and apoptosis of VSMCs enhances vascular calcification^15^. Differentiation of multipotent vascular stem cells to chondrogenic cells may also lead to vascular calcification and arterial stiffness^16^. Regardless of the process involved, the deposition of hydroxyapatite crystals requires the presence of collagen or elastic fibres that serve as solid-phase catalysts^12,17^. The proportion of smooth muscle cells and connective tissue in the medial layer of arteries (mostly elastic and collagen fibres) varies along the arterial vascular system, depending on the size and location of the vessel^18^. The medial layer of large arteries (thoracic and abdominal aorta, common carotid) contains abundant elastic fibres. As the vascular tree repeatedly branches into coronary and renal arteries amongst others, arterial walls exhibit more smooth muscle cells and fewer fibres^18^. In addition, as arteries become smaller, wall thickness gradually decreases. These structural changes of arteries could differently influence the occurrence of vascular calcification.

To study vascular calcification, in-vitro smooth muscle cell culture is often used^19^. This approach, however, cannot be used to investigate the susceptibility of different arteries to calcification. Rings sectioned from large arteries can successfully be cultured ex-vivo with maintained viability and structure over a long-term period^6,20^. Medial layer calcification of the ring is obtained with a calcifying medium supplemented with foetal bovine serum and phosphate^19,21^. Calcified vessels can also be obtained in animals with elastocalcinotic arteriosclerosis induced by hypervitaminosis D and nicotine, the VDN model^22^. In ex-vivo ring culture or in vivo calcification studies, arteries from distinct vascular beds, such as thoracic and abdominal aorta^2^, renal artery^3^, and intrarenal arteries^4,23^, can be analysed. The location and severity of vascular calcification may differently influence cardiovascular disease and mortality as suggested by the association of whole-body computed tomography scans of calcified atherosclerosis from five vascular beds with mortality in human^24^.

The aim of the present study was to investigate whether the location of the arteries influences their tendency to develop vascular calcification. To address this question, rings of various arteries isolated from the same animals were cultured in a calcifying medium and calcification was histologically determined. The influence of vessel injury on calcification was also evaluated. In addition, calcification of two different arteries, thoracic aorta and carotid was assessed in vivo using the vitamin D3-nicotine (VDN) rat model.

## Materials and methods

### Animals

For the ex vivo study of vascular calcification, four 6-week-old Wistar Han rats (SPF status, Charles River Laboratories) were housed in specific facilities (permit number D3417225) with a 12 h light/dark cycle in temperature-controlled conditions (22 ± 1 °C). Rats were fed normal rat chow (A04, SAFE) and tap water ad libitum. After one week of acclimatization, rats were anesthetized with isoflurane (2% in O_2_) and killed by decapitation before harvesting the vessels for ex vivo experimentations. Methods are more precisely described in a previous work^19^.

For the in vivo analysis of vascular calcification, eight six-week-old Wistar Han rats (Charles River Laboratories, L’Arbresle, France) received a single injection of vitamin D_3_ (300,000 IU kg^−1^, i.m.) and two gavages of nicotine (25 mg kg^−1^, 5 mL kg^−1^) on the same day (at 8 a.m. and 5 p.m.) to induce elastocalcinosis (VDN rats) as previously described^23^. Then rats were left quiet for four weeks until vessels were collected. Eight Wistar Han rats (age matched) were housed in the same conditions without receiving vitamin D_3_ and nicotine and formed the control group. Rats were randomly assigned to VDN and control groups at their arrival.

All animal experiments were performed according to the European Parliament Directive 2010/63/EU (N° CEEA-00322.03) and approved by the local ethics committee for animal experimentation of Languedoc-Roussillon (CEEA-LR, n°036, #17270). This study is reported in accordance with ARRIVE guidelines where applicable^25^.

### Vascular ring culture

Rings were isolated from arteries in aseptic conditions. The descending part of thoracic aorta, the abdominal aorta from diaphragm to iliac arteries, the left carotid artery and the left renal artery were gently cleaned of surrounding adipose tissue by careful dissection to avoid involuntary lesions. Descending thoracic aorta was divided into two parts, the proximal (upper part) and the distal (above diaphragm). Abdominal aorta was divided into four pieces, supraceliac, suprarenal, infrarenal, and suprailiac regions. Rings of 2–3 mm length were cut and placed by pairs in culture medium for 14 days. Some rings from the proximal or distal part of thoracic aorta and the carotid artery were cultured after the vessels were purposely injured by pinching and sliding forceps twice along the vessel in order to collapse the vessel lumen and induce intima-to-intima contact.

To induce calcification, rings were cultured in pairs in Dulbecco’s modified Eagle’s medium (DMEM, 4.5 g L^−1^ glucose) supplemented with 15% foetal calf serum, and containing 10 mmol L^−1^ sodium pyruvate, 3.8 mmol L^−1^ phosphate and 1.8 mmol L^−1^ calcium as described previously^19^. Some rings were placed in a control medium with 0.9 mmol L^−1^ of phosphate to measure the basal calcium content in the rings in absence of calcification. One ring of each well was used for the histological study, the second served for calcium content determination.

### Histological studies

For histological analysis, rings were fixed in 4% formaldehyde, paraffin embedded and cut into 5 µm-slices. After von Kossa staining (silver nitrate plus nuclear fast red), slices were photographed (TM300 NIKON microscope with a digital imaging system DXM1200 NIKON). Positive staining area was quantified in the medial layer and expressed as a percentage of the total medial area. The cross-sectional area (CSA, in mm^2^) of the rings was measured as the total surface area of the vessel on the Von Kossa slides.

For fibrosis determination, rings were stained with 0.1% picrosirius red and mounted in Eukitt medium (Sigma-Aldrich). Quantification in the medial layer stained was made in at least ten given fields per ring and expressed as a percentage of the total medial area.

Apoptotic nuclei in aortic wall of VDN rats were identified in thoracic aorta by TUNEL staining as previously described^19^.

All analyses were performed using image analysis software (ImageJ, National Institutes of Health, Bethesda, Maryland).

### Calcium content assay

The calcium content of vascular rings was determined by the o-cresolphthalein complexone method (Calcium Colorimetric Assay Kit Calcium, Biovision). Dried aortic rings were decalcified with 100 µL of 0.6 M of HCl, for 24 h and calcium content was measured in the supernatants. The calcium content was normalized to dry weight of the vascular ring and expressed as nanomoles of calcium per milligram of tissue (nmol mg^−1^).

### Statistical analysis

Values are presented as means ± SEM. For thoracic and abdominal aorta, 4 rings of each artery segment per rat were used for von Kossa staining area, and similarly for calcium content. For smaller vessels, the number of rings was limited by vessel length and the number of experimental units varied between 5 and 8 rings. For arteries isolated from VDN rats (and corresponding controls), experimental units were the 8 rats in each group. Student t-tests were used to compare the mean values between two groups. Alternatively, Welch t-tests were used when one group had a null variance. One-way ANOVA were used to compare three or more groups and followed by the Tukey Kramer multiple comparisons procedure evaluating all pairwise comparisons. We applied two-tailed tests with a type 1 error of 5%. p-values under 0.05 were considered statistically significant.

## Results

### Vascular calcification ex vivo in the different segments of the aorta

**Figure 1:**
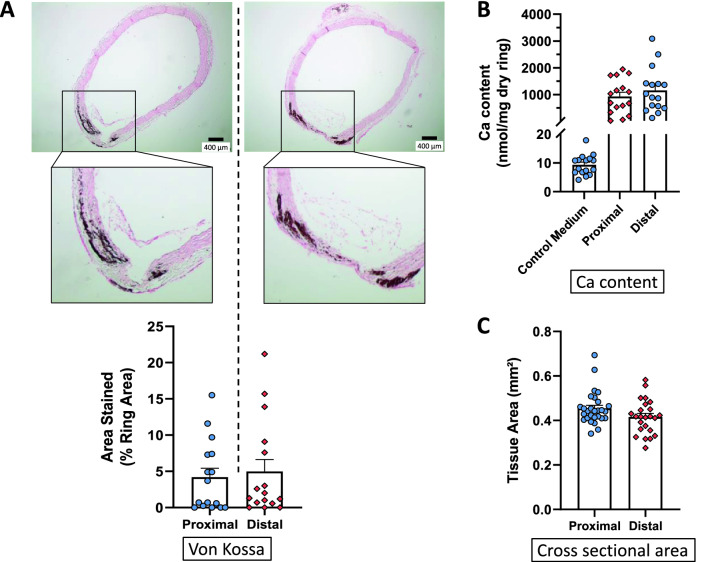
Ex vivo calcification of rings isolated from the proximal (upper) and distal (lower) part of the descending thoracic aorta. (**A**) Representative examples of von Kossa staining of rings, individual values and mean ± SEM of stained areas expressed as percentage of the media area, after 14 days of culture in a calcifying medium containing high phosphate (3.8 mmol L^−1^) and 15% foetal calf serum. Scale bar = 400 µm, original magnification × 40. (**B**) Calcium content of the rings (mean ± SEM) cultured in calcifying medium or in control medium. (**C**) Cross sectional area of the rings (mean ± SEM). No significant differences were detected between both parts of the thoracic aorta.

**Figure 2:**
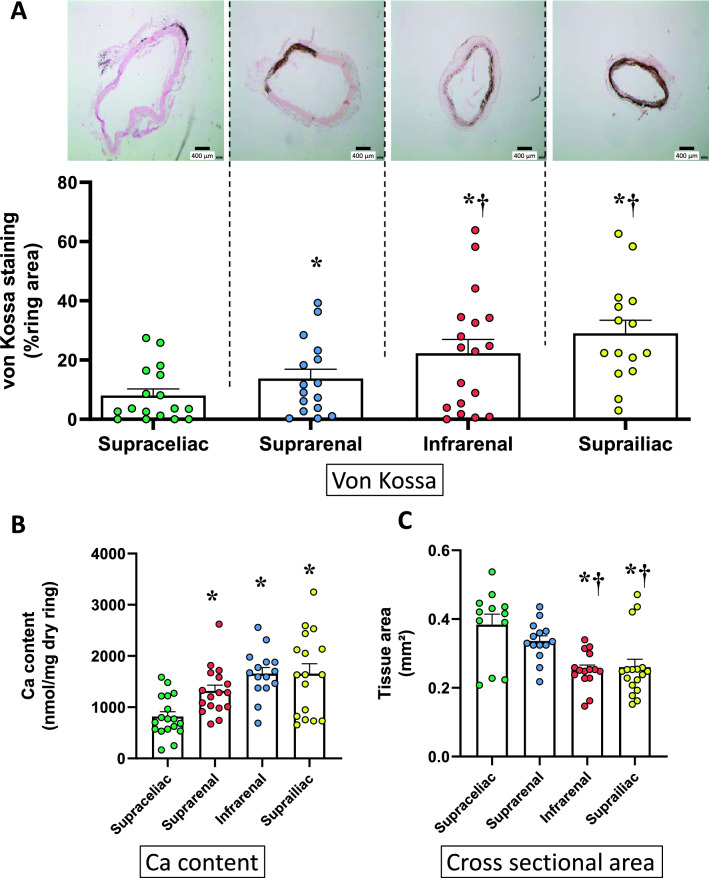
Ex vivo calcification of rings isolated from various parts of the abdominal aorta. Abdominal aorta (AA) was divided into four parts according to their location: supraceliac and suprarenal AA located, in the upper and lower part of the celiac trunk respectively (between diaphragm and renal arteries). Infrarenal and suprailiac segments are in the upper and lower part of the portion of AA between left renal artery and aortic bifurcation. (**A**) Example of von Kossa staining of rings and individual values and mean ± SEM of area stained after 14 days of culture in a calcifying medium containing high phosphate (3.8 mmol L^−1^) and 15% foetal calf serum. Scale bar = 400 µm, original magnification × 40. (**B**) Calcium content of the rings (mean ± SEM). (**C**) Cross sectional area of the rings (mean ± SEM). *p < 0.05 compared to supraceliac; ^†^p < 0.05 compared to suprarenal. No difference was found between infrarenal and suprailiac segments.

After 14 days of culture in calcifying medium with a phosphate donor and foetal calf serum, aortic rings presented focal von Kossa staining of the medial layer (Fig. 1A). Quantification of von Kossa stained area and calcium content in the proximal and distal parts of the thoracic aorta hardly differed from each other (Fig. 1A,B). The two thoracic aorta segments had similar CSA (Fig. 1C). In contrast, the different segments of the abdominal aorta displayed increasing von Kossa media staining from the supraceliac to the suprailiac portion (Fig. 2A). Calcium content gradually increased from the upper to the lower part of the vessel (Fig. 2B). CSA of the abdominal aorta gradually narrowed as presented on Fig. 2C. Of note, the two final portions of the abdominal aorta had similar calcium content and CSA.

### Vascular calcification ex vivo in different vascular beds and effect of injury

**Figure 3:**
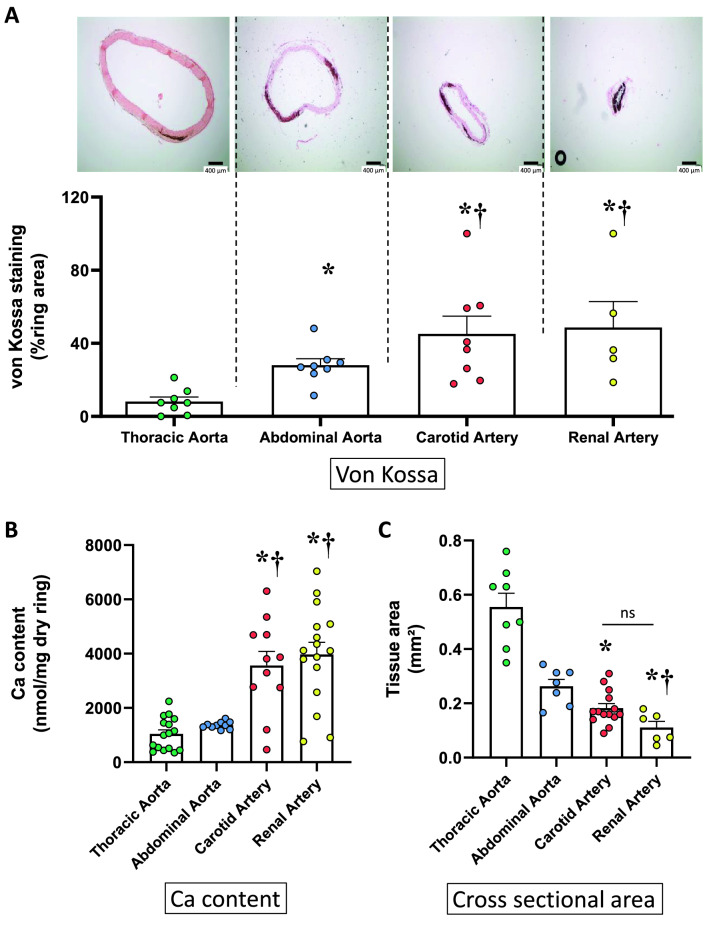
Ex vivo calcification of rings isolated from various vascular beds. (**A**) Example of von Kossa staining of rings (without injury) and individual values and mean ± SEM of area stained after 14 days of culture in a calcifying medium containing high phosphate (3.8 mmol L^−1^) and 15% foetal calf serum. Scale bar = 400 µm, original magnification × 40. (**B**) Calcium content of the rings (mean ± SEM). (**C**) Cross sectional area of the rings (mean ± SEM). *p < 0.05 compared to thoracic aorta; ^†^p < 0.05 compared to abdominal aorta. No difference was found between carotid and renal arteries; *ns* non-significant.

As illustrated in Fig. 3A, von Kossa stained area in intact rings (without injury) increased from the thoracic aorta to smaller arteries, as bifurcations occurred, and was similar in carotid and renal arteries. Calcium content was comparable in thoracic and abdominal aorta on the one hand, and in carotid and renal arteries on the other (Fig. 3B). CSA of arteries decreased with the distance to the heart (Fig. 3C).

**Figure 4:**
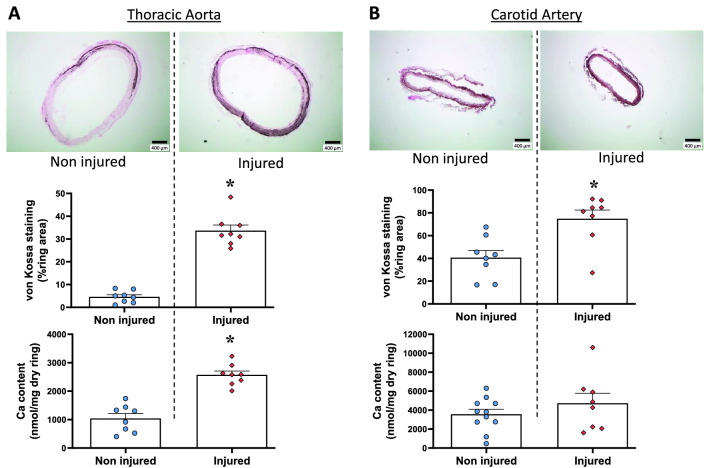
Ex vivo calcification of rings submitted to mechanical injury. Effect of injury on rings of (**A**) thoracic aorta and (**B**) carotid artery observed after 14 days of culture in a calcifying medium containing high phosphate (3.8 mmol L^−1^) and 15% foetal calf serum. Injuries were made by pinching and sliding forceps twice along the vessel in order to collapse the vessel lumen and induce intima-to-intima contact. Von Kossa staining is illustrated on micrographs and individual values and mean ± SEM of area stained and calcium content are presented. Scale bar = 400 µm, original magnification × 40. *p < 0.05 compared to non-injured corresponding rings.

Thoracic aorta and carotid arteries were voluntarily submitted to ex vivo injuries to determine the effect of sampling-induced potential lesions on rings calcification. As presented in Fig. 4A, both von Kossa stained area and calcium content of the rings increased in injured aortas. Similarly, carotid artery damage was associated with a doubling of the stained area, and with higher, yet non-significant, calcium content, compared to non-injured rings (Fig. 4B).

### Aorta and carotid artery calcification in VDN rats

**Figure 5:**
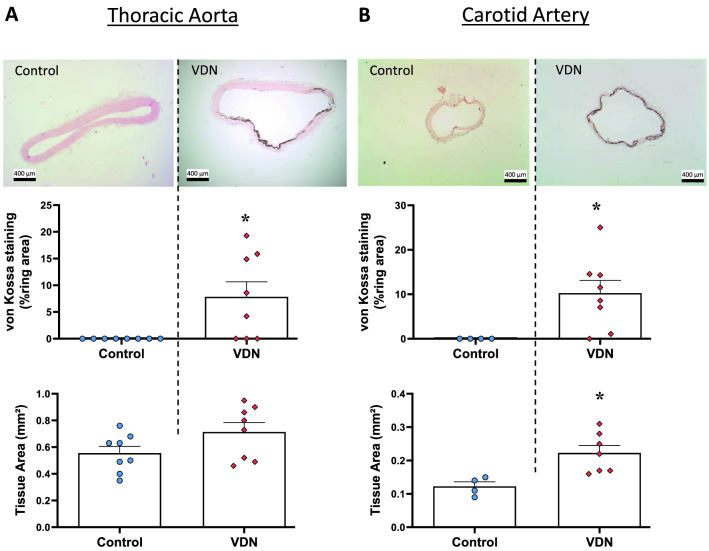
In vivo calcification of aorta and carotid artery of VDN rats. Von Kossa staining and cross sectional area of thoracic aorta (**A**) and carotid artery (**B**) obtained 4 weeks after vitamin D_3_ and nicotine (VDN) administration. Results are means ± SEM. Scale bar = 400 µm, original magnification × 40. *p < 0.05 compared to ring from control rats.

In vivo calcification was assessed on thoracic aortas and carotid arteries isolated from VDN rats. In control rats, von Kossa staining was absent in both arteries. In VDN rats, von Kossa stained area was clearly observed in these two arteries (Fig. 5). CSA was higher in VDN rats when compared to control animals and significance was only achieved for the carotid artery (p = 0.035) (Fig. 5).

**Figure 6:**
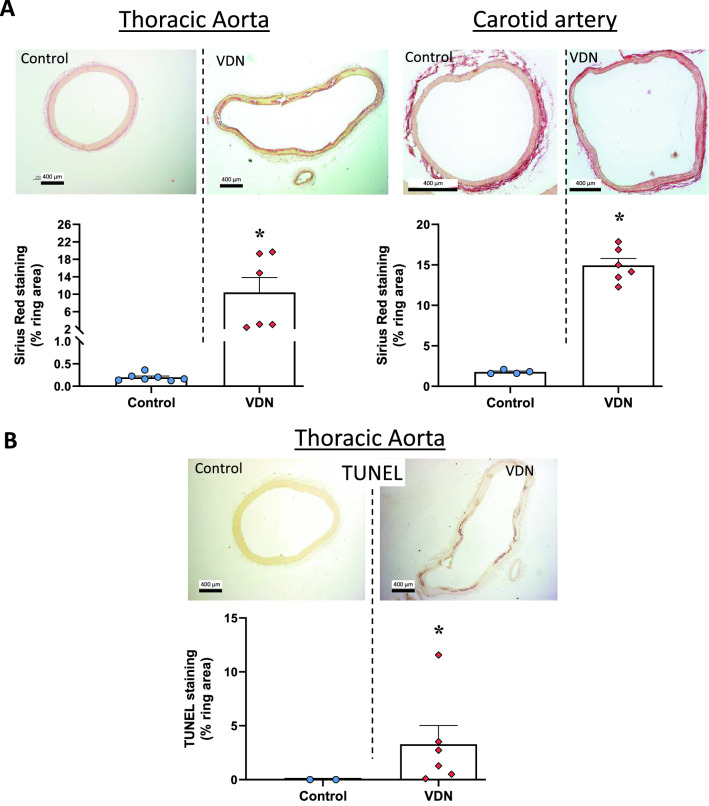
Fibrosis and apoptosis in arteries isolated from VDN rats. (**A**) Fibrosis was assessed by Sirius red staining of the aorta and carotid artery 4 weeks after vitamin D_3_ and nicotine (VDN) administration and in control rats (represented as mean area stained ± SEM). (B) Apoptosis was determined by TUNEL staining in the thoracic aorta and quantified (mean ± SEM). Scale bar = 400 µm, original magnification × 40. *p < 0.05 compared to ring from control rats.

Sirius red staining was performed to visualize fibrosis (collagen fibres) in the vessel wall. Stained area, excluding the adventitia, was markedly higher in both the aorta and the carotid artery from VDN rats (Fig. 6A). In the aorta, apoptosis determined by TUNEL staining was significantly higher in the VDN group (Fig. 6B).

## Discussion

The main objective of the present study was to evaluate whether different parts of the arterial network exhibit similar response to calcification. Our findings indicate that differences in susceptibility to calcification of the medial layer exist and can be revealed by ex vivo and in vivo approaches.

Firstly, it is worth noting that the ex vivo conditions used in this study were previously established in our lab^19^. Calcium deposit never occurred in intact vascular rings cultured in basal medium. Conversely, calcification was always observed in intact rings cultured in the high phosphate calcifying medium we developed. This is at variance with authors who previously concluded to an absence of calcium incorporation into arterial rings from control rats^20^ and healthy subjects^6^ cultured with high phosphate. The apparent discrepancy is very likely related to the presence of foetal calf serum in our calcifying medium. Indeed, we demonstrated that no calcium deposits were observed in absence of serum in the calcifying medium^19^, in line with observations of Lomashvili et al. and Shroff et al.^6,20^. In absence of serum, an intact inhibitory mechanism of hydroxyapatite formation in the vascular wall could be present^6^. Circulating compounds from foetal calf serum probably stimulate calcium deposition, as suggested by ring calcification observed after alkaline phosphatase addition to a serum-free, high phosphate medium^20^. Surprisingly, rings isolated from patients with chronic renal disease that are cultured in a serum-free medium with high phosphate/calcium levels displayed calcifications, while rings from healthy subjects did not^6^. These results suggest that ex vivo calcification requires a combination of high phosphate level and an additional factor which can be alkaline phosphatase or an unknown compound present in the serum.

Vessel integrity influences calcification. An extensive calcification occurs after injury as previously reported in mixed thoracic and abdominal aorta rings culture^20^. In the present study, voluntary injury of arteries before culture led to a marked increase in calcification of both thoracic aorta and carotid artery rings. Moreover, the calcification level achieved after injury in the carotid was striking (i.e. 70% of the area stained and more than 4500 nmol Ca/mg dry tissue). The effect of injuries was more marked in the smallest vessel than in the larger ones. Vascular lesions greatly increase calcification possibly explaining why rings from CKD patients known for frequent vascular diseases still calcified in serum-free calcifying medium^6^. Cautiousness is needed when dissecting arteries to isolate and culture rings to observe calcification. This also raises questions regarding the possible consequences of invasive procedures inducing vessel injuries.

Ex vivo calcification was explored in arteries of different sizes. CSA ranged from approximately 0.5 mm^2^ for the thoracic aorta to 0.1 mm^2^ for the renal artery. As the size of the vessel decreased, the calcium content and medial calcification increased. However, differences were not significant between the upper and lower parts of the thoracic aorta. Therefore, the whole thoracic aorta can be considered as a homogenous vessel regarding quantitative and qualitative ex vivo calcification assessments. On the opposite, rings from the abdominal aorta calcified differently depending on their location/size along the vessel. From the supraceliac to the suprailiac parts of the vessel, von Kossa staining increased regularly and significantly. The calcium load was confirmed by measurement of calcium content in these rings. Discrepancies between von Kossa staining and calcium content levels were occasionally observed, which are likely due to the technique. Calcium content was measured in the total aorta whereas the von Kossa staining was only considered in the media layer of aorta potentially leading to different results. In addition, von Kossa and calcium content cannot be performed on the same ring. Vascular calcification being a heterogeneous phenomenon, using different rings can also influence results. This is also relevant for in vivo results. Of note, comparable calcification indexes were observed for infrarenal and suprailiac segments which exhibited close cross-sectional area. The relationship between vessel size and induced calcification was corroborated by results obtained in the proximal and distal parts of the thoracic aorta and in non-aortic smaller vessels i.e. the carotid and renal arteries.

In agreement with the present ex vivo observations, a clearer calcification of the abdominal aorta compared to thoracic aorta was reported in in vivo models of calcification^2^. Therefore, thoracic and abdominal parts of the aorta should be considered as two different vascular beds, especially when their response to calcification is investigated.

Differences in the response to calcification may result from structural changes along the arterial tree. In aortas from Lewis rats, the number of elastic laminae decreases with distance from the heart, while the amount of smooth muscle and the relative wall thickness increases^2^. Consistently, in Wistar rats, the relative volume of smooth muscle cells and the wall to lumen ratio were higher in renal artery when compared to abdominal aorta^3^. Apatite deposition have been described on medial elastic fibres in the VDN model^26^ and it is associated to elastic fibres fragmentation^22^. More than the number of elastic laminae in the different vessels, elastin integrity seems critical^27^ and may vary in the different vessels investigated in the present study. Although the final site for calcification deposit is most likely the elastin layer, a body of evidence shows a central role of VSMCs osteoblastic transdifferentiation in vessel calcification^14,15,19^, and their relative increase in the media could favour calcification in small arteries. Moreover, VSMCs presents distinct features depending on their location and their various embryonic origin influences their ability to calcify^28^. Our results suggest that VSMCs from small arteries could be more prone to osteochondrogenic phenotype transition, and insufficiently express factors inhibiting vascular calcification. Furthermore, different hemodynamic characteristics (laminar versus turbulent flow, blood pressure amplitude, shear stress, etc.) on large and small arteries could influence elastin integrity and VSMCs phenotype resulting in different susceptibilities to vascular calcification^29^. Our findings indicate that ex vivo calcification is inversely proportional to arterial size and that location is important when interpreting findings.

In order to verify whether the relationship between vessel size and calcification can be observed in vivo, thoracic aorta and carotid artery were examined in a model of elastocalcinotic sclerosis obtained by hypervitaminosis D_3_ and nicotine (VDN)^23^. Similar to ex vivo results, calcification observed in vivo by von Kossa staining was doubled in a smaller artery (carotid) compared to thoracic aorta. Previous studies in the Lewis VDN rats compared changes in thoracic and abdominal aorta properties^2^. Ameer et al. reported higher stiffness and lamellae ruptures in the abdominal aorta than in the thoracic aorta, even if both vessels had similar calcium deposition levels^2^. Interestingly, in asymptomatic patients under 70 years of age, the prevalence of calcium deposition was higher in abdominal aorta, coronary and iliac arteries than in the thoracic aorta^24^. In addition to calcification, the VDN model was associated with more marked fibrosis in the carotid artery than in aorta. The concomitant observation of fibrosis and medial calcification was previously reported in ex vivo vascular ring culture^19^, diabetic mice^30^, hypertensive mice with elastic fibres disorder^31^ as well as in a rat model of calcification in chronic kidney disease^32^. On the other hand, a role for apoptosis in vascular calcification was evidenced in vascular smooth muscle cell culture^33^ and in the VDN rat model^34^. In the current study, TUNEL staining was increased in the thoracic aorta, confirming the association between calcification and apoptosis. Whether apoptotic cells are more present in smaller arteries where calcification is stronger remains to be confirmed.

The present study stressed the importance of size/location and integrity of a vessel, influencing its alteration in response to a disease-induced calcification. Therefore, ex vivo calcification of rings allows evaluation of regional specificity of vascular calcification which may be of importance in assessing the risk of cardiovascular and non-cardiovascular disease mortality^24^, particularly in chronic kidney disease^7^.

## Data Availability

The datasets generated and analysed during the current study are available from the corresponding author on reasonable request.
